# Direct intra-tumoral injection of zinc-acetate halts tumor growth in a xenograft model of prostate cancer

**DOI:** 10.1186/1756-9966-28-84

**Published:** 2009-06-17

**Authors:** Maulik R Shah, Christopher L Kriedt, Nathan H Lents, Mary K Hoyer, Nimah Jamaluddin, Claudette Klein, Joseph Baldassare

**Affiliations:** 1 Department of Otolaryngology, Saint Louis University, Saint Louis, Missouri, USA; 2 Saint Louis University Cancer Center, Saint Louis, Missouri, USA; 3 Department of Pharmacological and Physiological Sciences, Saint Louis University, Saint Louis, Missouri, USA; 4 Department of Sciences, John Jay College, City University of New York, New York City, New York, USA; 5 Department of Biochemistry, Saint Louis University, Saint Louis, Missouri, USA

## Abstract

Intracellular levels of zinc have shown a strong inverse correlation to growth and malignancy of prostate cancer. To date, studies of zinc supplementation in prostate cancer have been equivocal and have not accounted for bioavailability of zinc. Therefore, we hypothesized that direct intra-tumoral injection of zinc could impact prostate cancer growth. In this study, we evaluated the cytotoxic properties of the pH neutral salt zinc acetate on the prostate cancer cell lines PC3, DU145 and LNCaP. Zinc acetate killed prostate cancer cell lines *in vitro*, independent of androgen sensitivity, in a dose-dependent manner in a range between 200 and 600 μM. Cell death occurred rapidly with 50% cell death by six hours and maximal cell death by 18 hours. We next established a xenograft model of prostate cancer and tested an experimental treatment protocol of direct intra-tumoral injection of zinc acetate. We found that zinc treatments halted the growth of the prostate cancer tumors and substantially extended the survival of the animals, whilst causing no detectable cytoxicity to other tissues. Thus, our studies form a solid proof-of-concept that direct intra-tumoral injection of zinc acetate could be a safe and effective treatment strategy for prostate cancer.

## Background

In the United States alone, 200,000 men are diagnosed with prostate cancer each year and one out of six men will be diagnosed in their lifetime. As many as 30,000 men die from this disease each year in the US, making prostate cancer the second biggest cancer killer of men, behind lung cancer[1]. However, several distinct features of the prostate gland open up unique opportunities for treatment of this cancer. First, the prostate is a nonessential organ, often making complete surgical resection a viable option, albeit one with permanent unpleasant side effects for the patient. Secondly, during early phases of the disease, the malignant prostatic lesions tend to remain focal and restrictively localized to the prostate gland itself. This, combined with the anatomic accessibility of the prostate gland, makes direct intra-tumoral injection of carcinotoxic and carcinostatic agents a real possibility for effective and relatively noninvasive treatment[2]. In this study, based in part on promising *in vitro *results from our laboratory, we explore the effectiveness of direct intra-tumoral injection of zinc acetate into malignant prostatic tumors.

Zinc is the most abundant trace element in the human body and is vital for the function of many enzymes and proteins in all cells and tissues of the body. There are over 300 zinc-dependent enzymes and zinc is required for the formation of the zinc-finger motif that is an essential component for nearly all transcription factors and many other proteins that bind nucleic acids[3]. It has long been known that chronic insufficient dietary zinc leads to many debilitating developmental defects, but emerging evidence now links marginally deficient zinc consumption, such as that which affects more than 10% of the US population, to such diseases as anorexia nervosa, Ahlzeimer's Disease, and cancer. Several studies have found that men who consume below the USDA recommended daily allowance (RDA) of 11 mg/day are at increased risk of developing prostate cancer[4]. Conversely, other studies have shown that high-dose supplements of zinc can increase the risk of prostate cancer[5]. Thus, the role of dietary zinc in the predisposition to prostate cancer requires further study.

The relationship between dietary zinc and prostate cancer likely stems from the vital role that zinc plays in prostate function. Zinc is known to accumulate in the prostate, and this gland typically harbors the highest concentration of zinc in the body[6]. This is because the secretory cells of the prostate require high levels of zinc to inhibit the enzyme m-aconitase, which normally functions to oxidize citrate during the Krebs cycle. Because citrate is a principle component of seminal fluid, prostate secretory cells do not complete the oxidation of citrate in the mitochondria and the zinc-mediated inhibition of m-aconitase is crucial for the accumulation of citrate in these cells, and thus the subsequent secretion of citrate into seminal fluid[7]. The accumulation of zinc in the prostate epithelium is accomplished by the zinc transporter ZIP1, which is highly expressed in normal prostate tissue[8].

Because zinc is thus antagonistic to the synthesis of ATP in the cells of the prostate gland, it is not surprising that both ZIP1 expression and the accumulation of zinc are markedly attenuated in a cancerous prostate [9]. [10]. Indeed, ZIP1 is considered a prostate tumor suppressor as the inhibition of its function is requisite for malignant transformation, and prostatic zinc levels have shown an inverse relationship with tumorigenicity [11]. Thus, the restoration of zinc levels in prostate cancer cells is a logical strategy for clinical treatment. Further, zinc has been shown to be required for mitochondrial apoptogenesis in prostate cells *in vitro *[12], and infusions of moderate doses of zinc reliably lead to apoptosis of prostate cancer cell lines [13]. This has led to the hypothesis that clinical administration of zinc could be an effective chemotherapeutic for prostate cancer. However, studies of zinc dietary supplementation for cancer prevention have had mixed results [14,15].

Recently, vascular delivery of zinc was evaluated as a potential treatment in a mouse model of prostate cancer [6]. Although an increase in apoptosis was observed in the prostate cancer xenografts of the mice receiving high doses of zinc, there was little effect on the overall growth and aggressiveness of the prostate tumors themselves. Because ZIP1 function is known to be impaired in prostate cancer cells, we presume that there was limited homing of zinc to the prostate cancer xenografts. Thus, we reason that a localized infusion of zinc, and thus a greater local concentration, could circumvent the reduced ZIP1 activity and allow greater bioaccumulation of zinc in the diseased prostate. This is important because intravenous doses of zinc are limited due to the cellular toxicity of this heavy metal at super-physiologic concentrations.

In this study, we hypothesize that the direct intra-tumoral injection of zinc could be a safe and efficacious treatment for prostate cancer. To our knowledge, this is the first examination of intra-tumoral zinc delivery as a treatment strategy for prostate cancer, and we feel that these data form powerful preliminary evidence indicating that such a minimally invasive strategy could be efficacious. Furthermore, because of the preferential accumulation of zinc in prostate tissue, it is conceivable that such a strategy could be entirely free of the debilitating and dose-limiting side effects typical of other cancer chemotherapeutics.

## Methods

### Cell lines

PC3, DU148, LNCaP cells were originally obtained from ATCC (Rockville, Maryland, USA). Cells were maintained at 37°C, 5% CO_2 _and 95% humidity in DMEM (CellGro, Herndon, Virginia, USA) supplemented with 10% (v/v) heat inactivated fetal bovine serum (BioWhittaker, Walkersville, Maryland, USA), 2 mM L-glutamine and 100 units/ml penicillin and 1000 ug/ml streptomycin (Invitrogen, Carlsbad, California, USA).

### Animals

NOD/SCID mice at 8 weeks of age were purchased from Charles River Laboratories (Wilmington, Massachusetts, USA) and were housed at the Saint Louis University comparative medicine facility. Animals were allowed to acclimate for 2 weeks prior to experimentation. The animals were under the care of a staff veterinarian and managed in accordance with the National Institutes of Health Guide for the Care and Use of Laboratory Animals.

### Xenografts

PC3 cells grown to 70% confluence were harvested and injected in the dorsum of animals subcutaneously. Each inoculum consisted of 100 μL of cell suspension at a concentration of 10^7 ^cells/ml in phosphate-buffered saline. Tumors were allowed to grow to a size of 300 mm^3 ^prior to intra-tumoral injection. Tumors were injected with 200 μL of 3 mM zinc acetate solution every 48 hours. Tumors were measured every 2–3 days with digital calipers. Tumor volume was determined using the following formula: Volume = Length × Width^2^.

### Zinc Measurements

Zinc was quantified in serum and tissues using the TSQ fluorophore (Invitrogen, Carlsbad, California, USA). 50 mM TSQ was prepared in 10 mM Tris buffer (ph = 8.0). TSQ was added to samples and standard zinc solutions to a final concentration of 10 μM in black round-bottom 96 well plates. Endpoint fluorescence was read on a Spectfluor with excitation wavelength of 360 nm and emission wavelength of 535 nm. Tissue zinc levels were measured similarly, after weighing and homogenizing tissue in water by repeated freeze/thaw cycles.

### MTT Assay

Cell viability was determined via MTT assay. Briefly, media was aspirated from cells grown in 6 well plates and 1 ml of MTT (1 mg/ml) solution was added. After 1 hour incubation, MTT solution was aspirated and 0.04 N HCL was added to solubilize the cells and absorbance at 540 nM was measured.

## Results

Zinc has been shown to be cytotoxic to a variety of cancer cell lines [16-18]. In these studies, different formulations of zinc have been utilized. Unfortunately, *in vivo *measurements regarding the bio-pharmocokinetics of these different zinc salts are lacking. For this study, we have selected zinc acetate as it is pH neutral in aqueous solution with minimal effect on osmalarity, relative to other formulations of zinc. Cytotoxic effects of zinc acetate have not been reported.

In order to examine the general effectiveness of zinc in inducing cell death in prostate cancer cells, we selected three cell lines with distinct properties, representative of the distinct forms in which prostate cancers emerge. For example, PC3 and DU145 cells are androgen-independent, while LNCaP cells are androgen-dependent[19]. The molecular pathways associated with carcinogenesis vary as well between these cell lines[20] as determined by gene expression analysis. For example, PSA is upregulated in LNCaP but not expressed in PC3 or DU145. Using markedly different prostate cancer cell lines allowed us to analyze the effect of zinc irrespective of underlying pathways of transformation.

### Induction of apoptosis of prostate cancer cells by zinc

**Figure 1:**
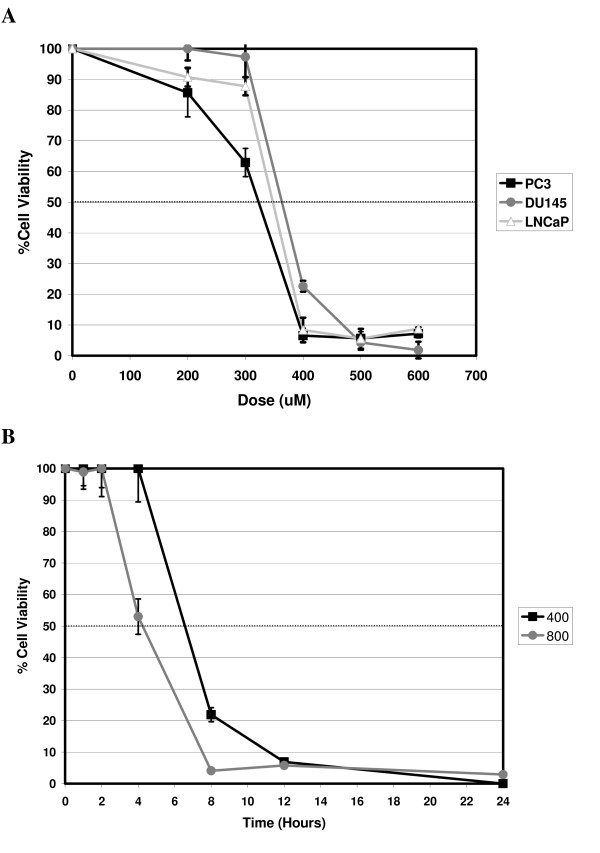
**Kinetics and Toxicity of Zinc Acetate on Prostate Cancer Cell Lines**. Prostate cancer cell lines (Panel A: PC3, DU145, and LNCaP; Panels B and C: PC3) were treated with the indicated concentrations of zinc acetate for either 18 hours (A) or indicated length of time (B and C). Data represent mean cell viability as assessed by MTT assay (n = 3 independent cell populations) and error bars represent standard deviation.

In figure 1, we show that treatment with zinc acetate leads to widespread cell death within 18 hours in three different prostate cancer cell lines (figure 1A). Importantly, cell death is sharply dose-dependent over a broad range from 100–600 μM and the cytotoxicity curves indicate that 300–400 μM zinc acetate, depending on cell line, is effective at inducing cell death in ~80% of the cell population within just 18 hours (figure 1A). Having established that zinc acetate has a rapid cytotoxic effect on prostate cancer cell lines, we next established the time course of cell killing *in vitro*. Although only data for PC3 cells are shown, for all three cell lines, 400 μM zinc acetate induced cell death quite rapidly, with 50% cell death occurring by 6 hours (figure 1B and data not shown). By 24 hours, greater than 95% of the cells had perished. Interestingly, zinc dose had minimal effect on the kinetics of cell death, as doubling the dose to 800 μM zinc only reduced the EC50 by approximately 90 minutes (figure 1B).

Although maximal cytotoxicity is seen within 24 hours with doses of 400 μM zinc or higher, we reasoned that longer incubations with lower doses of zinc might also have a cytotoxic effect on prostate cancer cells. Thus, we next evaluated zinc-induced cytotoxicity in PC3 cells at lower doses and found that, surprisingly, at each dose, maximum cell death again occurred by 24 hours with little further cell death thereafter (data not shown). However, prolonged exposure to zinc, even at the lowest dose of 100 μM, has a cytostatic effect: cellular proliferation halted and the number of cells remained constant over time (data not shown). Indeed, this cytostatic effect of prolonged exposure to zinc was observed at all doses explored in this study.

### Effect of Zinc Acetate on PC3 Xenograft Growth

Given these promising *in vitro *results, we next examined whether zinc treatments could affect prostate cancer cells *in vivo*. To that end, we established a human prostate cancer xenograft model by injecting a bolus of PC3 cells subcutaneously into the dorsal region of SCID mice. To date, detailed toxicity reports of zinc acetate in mice are lacking. However, experiments with mice have revealed an LD50 of approximately 50 mg/kg for zinc chloride [21]. Because the maximal tolerable dose of zinc acetate has not been established and given that chronic liver changes were observed at the LD50 dose, we elected to use a dose that approximated one-eighth of the LD50, 200 μL of 3 mM zinc acetate. In a pilot study, we observed that a single dose of zinc acetate had no measurable effect on tumor growth (data not shown). In addition, because previous studies have established that zinc is rapidly distributed in total body water and cleared by renal filtration within 24 hours[22], we elected to administer repeated doses of zinc acetate in 48 hours intervals in order to establish a chronic treatment protocol, while limiting untoward zinc bio-toxicity and stress to animals due to the repeated anesthesia and injection.

**Figure 2:**
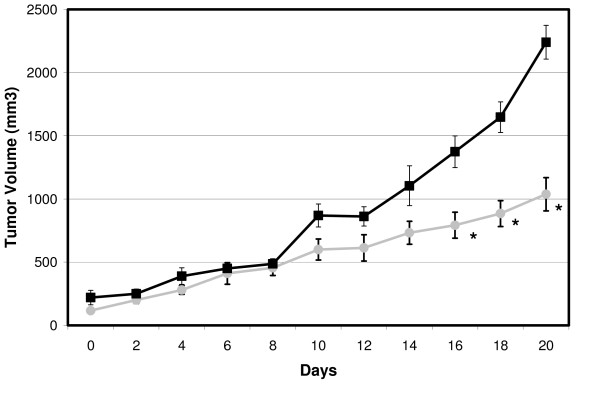
**Effect of Direct Intra-Tumoral Zinc Injection on PC3 Growth**. Prostate cancer cell xenografts were placed into SCID mice and allowed to grow to a size of 300 mm^3^. Every 48 hours for 14 days, mice were then anesthetized and injected with 200 μL of either saline (black squares) or 3 mM zinc acetate (grey circles). Tumor size was measured at the indicated intervals. Error bars represent average size for 10 mice and "*" represents significance at p < 0.05 by ANOVA.

When the prostate tumor xenografts reached 300 mm^3^, treatments were begun: 200 μL of 3 mM zinc acetate by direct intratumoral injection every 48 hours for a period of two weeks. We selected this somewhat large tumor size for both ease of intratumoral injection, and also for greater accuracy and consistency when using size as an outcome measure. Figure 2 demonstrates the effect of the zinc injections on tumor growth and it is immediately clear that intratumoral injections of zinc have a profound negative effect on growth of the tumor xenografts. The injection of zinc dramatically halts the aggressive growth of PC3 xenografts and, importantly, the growth arrest persists after the injection schedule is terminated on the fourteenth day (figure 2). Importantly, the growth of xenografts was unaffected by the anesthesia and injection procedure *per se *as vehicle-injected tumors display growth kinetics indistinguishable from that of non-injected xenografts.

### Bioavailability of zinc following intra-tumoral injection

**Figure 3:**
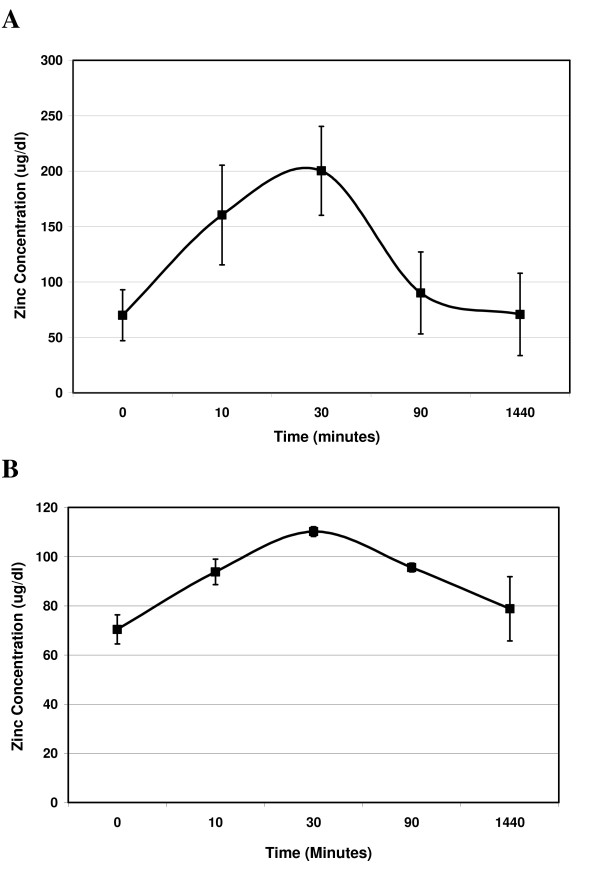
**Serum Zinc Levels after Subcutaneous or Intratumoral Zinc Injection**. Serum levels were measured at the indicated times following either a subcutaneous (A) or an intratumoral (B) single 200 μL injection of 3 mM zinc acetate. Data is presented as an average and errors bars indicate the standard deviation of four mice (n = 4).

Because of the promising results of arrested prostate cancer cell growth following zinc injection, we next turned our attention to the biodistribution of the zinc in this context. We began with simple subcutaneous injections of zinc acetate in otherwise un-treated SCID mice and found that single injections of zinc result in a rapid increase in serum zinc levels as early as 10 minutes after administration (figure 3A). However, serum zinc levels peak in 90 minutes and return to normal physiological levels within 24 hours (figure 3A). We next examined the pharmacokinetics of intra-tumoral injection of zinc acetate into our prostate cancer xenografts model. The resulting kinetics of zinc distribution are similar: serum zinc levels rise quite rapidly after tumor injection, reaching a maximum within 90 minutes, followed by a steady decline to baseline levels within 24 hours (figure 3B). A significant difference is that peak serum zinc levels are considerably less when injected into tumors then subcutaneously indicating either slower release from tumor tissue or significant uptake into tumor tissue.

**Figure 4:**
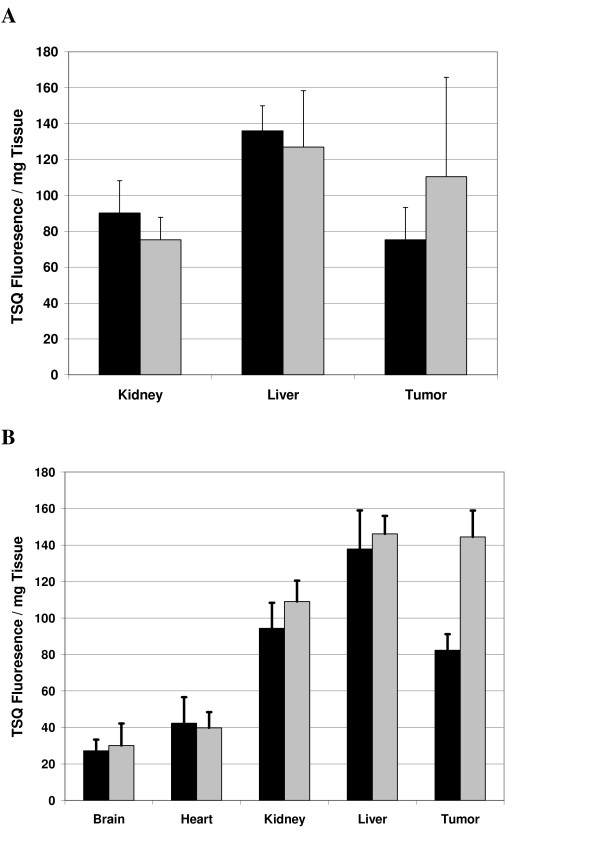
**Tissue Zinc Concentration After Acute or Chronic Zinc Administration**. Levels of zinc were measured in specific tissues following either a single (A) or chronic (B) 200 μL injections of 3 mM zinc acetate. Data is presented as an average and errors bars indicate the standard deviation of four mice (n = 4).

We also sought to examine the homing of zinc to different tissues, following a single intra-tumoral injection. As shown in figure 4A, although the liver displayed the greatest concentration of zinc, there is no significant difference in zinc levels after zinc administration, although we observed considerable variability between animals. Similarly, there appears to be a reproducible but statistically insignificant accumulation of zinc within the xenograft tumors, even after a single administration (figure 4A). We then extended these observations to conditions of chronic zinc administration and found that our intratumoral zinc injection protocol results in a substantial increase in zinc levels within the tumor xenograft cells, but not in any brain, heart, kidney, or liver (figure 4B). This confirms our supposition that intra-tumoral injection allows for a much higher local concentration of zinc, which in turn may overcome impaired zinc import and thus, increased partitioning of therapeutic zinc into the diseased prostate tissue.

### Zinc Biosafety

Over the course of our experiments, we were able to limit prostate xenograft growth over a period of two weeks. Even after zinc administration was discontinued, tumor growth was slower than in control animals (figure 2). Importantly, at the dosage delivered to the animals, we did not observe any evidence of biotoxicity during the treatment protocol and no animal death was recorded. Further, a blinded pathologist performed a full post-mortum histological analysis of tissues and uncovered no evidence of tissue toxicity in the animals enrolled in the zinc treatment protocol (data not shown). Liver changes reported by others at the LD50 level were not seen with our substantially lower dosage even with the chronic administration schedule.

### Survival of Animals following treatment of prostate cancer xenografts with zinc

As a final measure of the potential usefulness of zinc as a component of prostate cancer chemotherapeutics, we assayed the ability of the intra-tumoral zinc injection protocol to extend the life of animals in our prostate cancer xenograft model. Because they are growing subcutaneously rather than orthotopically xenograft tumors may grow to significant size without causing animal death. For humane reasons, a scoring system was established to assess animal welfare and animals not able to meet two requirements were euthanized. The scoring system consisted of the following: 1. Maintenance of normal weight (Weight loss > 12%); 2. Normal ambulation; 3. Normal grooming; 4. Normal feeding. Importantly, the decision to remove an animal from the protocol due to extreme tumor burden was made by an animal care technician unaware of the treatment group of the particular animal at the time of the decision.

**Figure 5:**
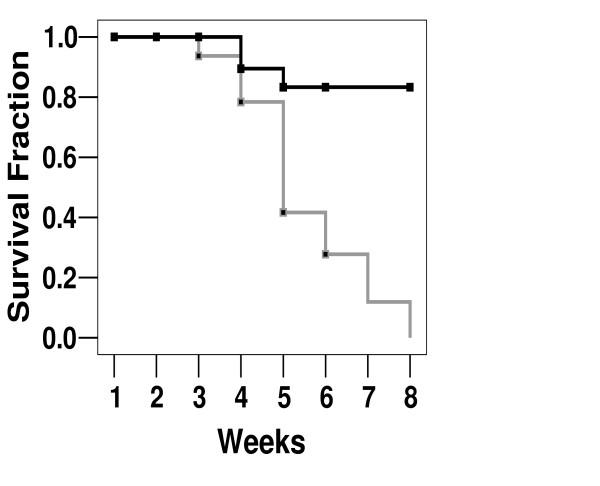
**Effect of Intra-Tumoral Zinc Injection on Survival**. Prostate cancer cell xenografts were placed into SCID mice and allowed to grow to a size of 200 mm^3^. Every 48 hours for 14 days, mice were then anesthetized and injected with 200 μL of either saline or 3 mM zinc acetate. Mice were evaluated by a blinded technician and sacrificed when tumor burden reached predetermined criteria for humane maintenance and care. Difference in mean survival between treatment and control groups was significant (p < 0.002) by Kaplan-Meier Survival Analysis.

Thus, humane removal of an animal from the protocol was recorded as a death event, and with these data we evaluated survival. As seen in figure 5, intra-tumoral injection of zinc acetate significantly extended the lifespan of animals in this xenograft model of prostate cancer. Dramatically, although the treatment protocol extended for only two weeks, the enhanced survival of animals in the zinc treatment group was persistent for several weeks beyond (figure 5). In the control group, all animals had succumbed to the debilitating effects of the growing tumor within eight weeks of the beginning of the treatment protocol. However, in the same time period, 80% of those treated with zinc acetate injections remained alive (figure 5). This dramatic result was significant (p = 0.002) by Kaplan-Meier Survival Analysis and revealed the intra-tumoral injection can halt the growth of prostate cancer *in vivo *with marked in gains in survival.

## Discussion

Prostate cancer represents a unique clinical problem with respect to treatment options. 90% of men will present with localized disease [23]. For these men, the current treatment paradigm is prostatectomy or radiotherapy. For men with advanced disease, androgen therapy offers the best opportunity for long term survival. However, treatment may be limited by the androgen responsive nature of the tumor. Given the age at which many men present with prostate cancer and the slow growing nature of this cancer, in many cases, the treatment options may have equivalent morbidity in comparison to the cancer itself. Hence, less invasive methods of treatment with fewer side effects would be very advantageous for men presenting with localized disease.

There is much to suggest that treatment with zinc has real clinical potential. It is solidly established that reduced intracellular zinc levels are necessary for maintaining the malignant phenotype of prostate cancer cells [24] and that malignancy and tumor aggressiveness are inversely proportional to tumoral zinc levels [25]. Thus, the current paradigm for zinc in prostate cancer suggests that loss of intracellular zinc is vital to the transformation of normal prostate tissue into cancerous prostate tissue, likely due to the metabolic effects of zinc in the Krebs cycle. That is, because zinc inhibits m-aconitase, loss of zinc allows for greater energy utilization, supporting the substantially increased cellular metabolism that is necessary for rapid proliferation [26].

Because systemic (i.e. intravenous) injection of zinc has limitations and is poorly targeted to diseased prostate, in this study we evaluated whether increasing zinc bioavailability through direct injection into tumors would impact prostate cancer malignancies. Although repeated intratumoral injections may not be a desirable treatment modality for human prostate cancer patients, we have provided proof of concept that increase of intraprostatic zinc can effectively moderate prostate tumor growth.

In our *in vitro *experiments, we have shown that increasing zinc in the microenvironment to 200–600 μM can cause rapid prostate cancer cell death. Cell death was independent of the mechanism of molecular carcinogenesis and independent of androgen sensitivity. Others have reported that the mechanism of zinc associated prostate cancer cell death is apoptotic with a shift in Bax/BCL2 ratios[27] and the morphological changes seen in our studies are consistent with apoptotic cell death. Cell death was also quite rapid indicating that prolonged exposure is not necessary for zinc effects on prostate cancer cells.

Human physiological serum zinc levels are approximately 70–100 μg/dL. This represents total zinc and not any particular salt form. As such, it is difficult to reconcile levels with molar doses used in our *in vitro *studies, but it is clear that our *in vitro *doses could not be achieved systemically in a whole animal without undue toxicity. However, we hypothesized that, given the rapid nature by which zinc-mediated cell death occurs in prostate cancer cells, the local microenvironment could be altered to a level sufficient to impact tumor growth whilst avoiding widespread toxicity. Thus, in an attempt to maximize the anti-tumor effect and minimize the biotoxicity, we selected a dose that was approximately 8-fold less than the LD50 toxic dose reported for rodents. Based on the fact that we had no observed tissue biotoxicity, future studies could determine the maximum tolerable dose for direct zinc administration.

## Conclusion

Our results showed that despite rapid dissipation of zinc into total body water there was a local effect of diminishing tumor growth over time. Although our administration schedule is an impractical method for the treatment of local disease in humans, our studies have established that administration of zinc in the tumor microenvironment can have a potent anti-tumor effect. Direct injection into tumors did result in increasing tumor tissue zinc levels and altered growth over time, an effect that persisted long after zinc injections were ceased. Our data indicate that methods to increase zinc in the prostate tumor microenvironment could be useful as a way of modulating growth of localized disease. Given rapid physiological clearance of zinc, the use of zinc would likely have limited systemic toxicity. Consequently, injection of biogels or depot formulations of zinc may be an alternative strategy to increasing intraprostatic zinc resulting in anti-tumor effect with limited biotoxicity.

## Competing interests

The authors declare that they have no competing interests.

## Authors' contributions

MRS, CK and JB contributed equally to the design and implementation of the study. MRS was responsible for all statistical analysis. MRS and NHL drafted the manuscript. CLK and MKH equally contributed to carrying out all *in vitro *zinc toxicity studies. CLK performed all of the animal experimentation. NJ and CLK performed all zinc level determinations. All authors have read and approved manuscript.
